# Optimal timing of rest perfusion with regadenoson stress testing - normal volunteer study of quantitative MRI perfusion

**DOI:** 10.1186/1532-429X-13-S1-P128

**Published:** 2011-02-02

**Authors:** Sujethra Vasu, W Patricia Bandettini, Li-Yueh Hsu, Peter Kellman, Joel Wilson, Steve Leung, Sujata M Shanbhag, O Julian Booker, Christine Mancini, Jennifer Henry, Tracy Lowrey, Andrew E Arai

**Affiliations:** 1 National Institutes of Health, Bethesda, MD, USA

## Introduction

Many MRI perfusion protocols perform rest imaging a few minutes after stress imaging. Regadenoson is a new, selective Adenosine-2A receptor agonist used for myocardial perfusion imaging. The purpose of this study was to assess how well rest perfusion imaging 20 minutes after regadenoson stress reflects true baseline rest perfusion.

## Hypothesis

“Rest perfusion” performed 20 minutes after regadenoson stress and reversal with aminophylline is higher than rest perfusion before administration of regadenoson.

## Methods

Seventeen healthy normal volunteers with Framingham score less than 1% underwent vasodilator stress testing with regadenoson using a SSFP sequence. Rest imaging (Rest 1) was performed initially. Twenty minutes later, regadenoson 400mcg was administered as a 10 second injection with saline flush. Stress imaging was done 70 seconds after the injection. All volunteers received aminophylline 100mg. A second rest perfusion (Rest 2) imaging was done 20 minutes later. Myocardial blood flow (MBF) in ml/min/g and myocardial perfusion reserve (MPR) were quantified using a fully quantitative model constrained deconvolution (MCD).

## Results

**Figure 1:**
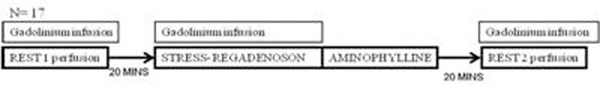
Study design

**Figure 2:**
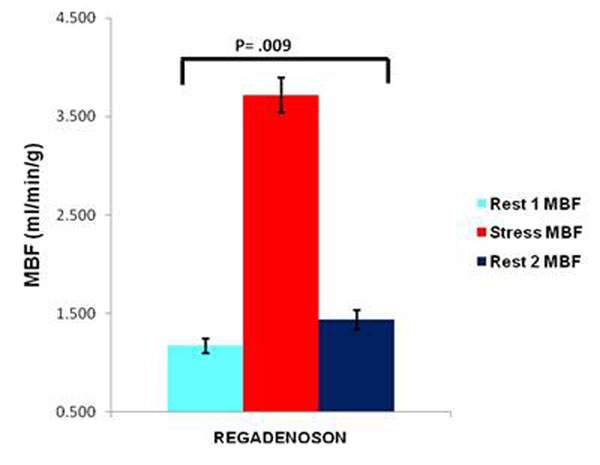
Rest 1, Stress and Rest 2 MBF

Rest 2 MBF (mean ± standard error) done 20 minutes after regadenoson stress was 1.43 ± 0.10, higher than rest perfusion done before regadenoson administration (Rest 1) is 1.18 ± 0.07, p=.009. The stress MBF is 3.72 ±0.18. Using the rest perfusion after regadenoson as true resting perfusion, leads to a 15% underestimation of MPR (2.75 ± 0.21 Stress/Rest 2 vs. 3.33 ± 0.22 Stress/ Rest1, p=0.005). Figures 1 and 2.

## Conclusions

Rest perfusion done 20 minutes after regadenoson 400 mcg has not returned to baseline resting myocardial perfusion despite reversal with 100mg of aminophylline. This leads to underestimation of MPR. Regadenoson has a longer duration of effect which might be less suitable for rest perfusion done 20 minutes after stress.

**Figure 3:**
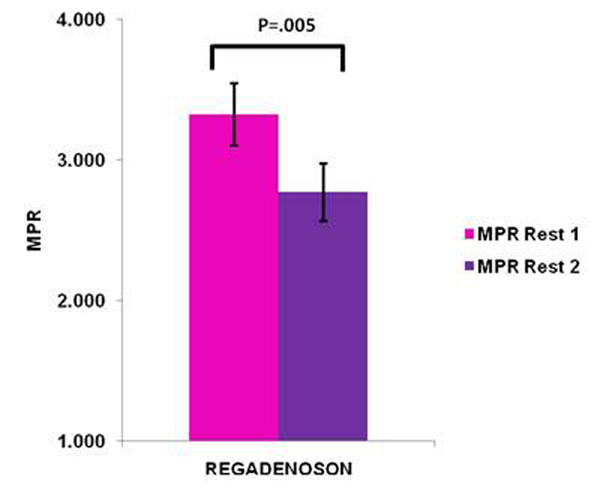
Comparison between MPR based on Rest 1 and Rest 2 MBF.

